# bioTCIs: Middle-to-Macro Biomolecular Targeted Covalent Inhibitors Possessing Both Semi-Permanent Drug Action and Stringent Target Specificity as Potential Antibody Replacements

**DOI:** 10.3390/ijms24043525

**Published:** 2023-02-09

**Authors:** Jay Yang, Yudai Tabuchi, Riku Katsuki, Masumi Taki

**Affiliations:** 1 Department of Engineering Science, Graduate School of Informatics and Engineering, University of Electro-Communications (UEC), 1-5-1 Chofugaoka, Chofu 182-8585, Japan; 2 School of Medicine and Public Health, University of Wisconsin, Madison, WI 53706, USA; 3 Department of GI Surgery II, Graduate School of Medicine, Hokkaido University, Sapporo 068-8638, Japan; 4 Institute for Advanced Science, UEC, Chofu 182-8585, Japan

**Keywords:** covalent aptamer, protease/nuclease resistance, warhead, middle-molecule covalent drug, peptide/oligonucleotide therapeutics, reactivity and affinity-based co-selection, reversing adverse drug effects (ADEs), matchmaking microenvironment, pharmacokinetic parameter (*k*_inact_/*K*_I_), quiescent affinity label

## Abstract

Monoclonal antibody therapies targeting immuno-modulatory targets such as checkpoint proteins, chemokines, and cytokines have made significant impact in several areas, including cancer, inflammatory disease, and infection. However, antibodies are complex biologics with well-known limitations, including high cost for development and production, immunogenicity, a limited shelf-life because of aggregation, denaturation, and fragmentation of the large protein. Drug modalities such as peptides and nucleic acid aptamers showing high-affinity and highly selective interaction with the target protein have been proposed alternatives to therapeutic antibodies. The fundamental limitation of short in vivo half-life has prevented the wide acceptance of these alternatives. Covalent drugs, also known as targeted covalent inhibitors (TCIs), form permanent bonds to target proteins and, in theory, eternally exert the drug action, circumventing the pharmacokinetic limitation of other antibody alternatives. The TCI drug platform, too, has been slow in gaining acceptance because of its potential prolonged side-effect from off-target covalent binding. To avoid the potential risks of irreversible adverse drug effects from off-target conjugation, the TCI modality is broadening from the conventional small molecules to larger biomolecules possessing desirable properties (e.g., hydrolysis resistance, drug-action reversal, unique pharmacokinetics, stringent target specificity, and inhibition of protein–protein interactions). Here, we review the historical development of the TCI made of bio-oligomers/polymers (i.e., peptide-, protein-, or nucleic-acid-type) obtained by rational design and combinatorial screening. The structural optimization of the reactive warheads and incorporation into the targeted biomolecules enabling a highly selective covalent interaction between the TCI and the target protein is discussed. Through this review, we hope to highlight the middle to macro-molecular TCI platform as a realistic replacement for the antibody.

## 1. Introduction

### Targeted Covalent Inhibitors (TCI) as Potential Antibody Replacements

Just 37 years since the first approval of an antibody for human use in 1986, antibody-based therapy [1,2] is the fastest growing drug modality now with over 160 approved antibodies (https://www.antibodysociety.org/resources/approved-antibodies/, accessed on 20 December 2022) against 91 drug targets for clinical usage world-wide for a variety of clinical indications [3]. Clinicaltrials.gov (accessed on 30 October 2022) lists 1504 ongoing actively recruiting clinical trials with various antibodies, suggesting that the antibody therapy boom continues unabated, and the currently $186 billion market is estimated to reach $445 by 2028 (https://www.gminsights.com/industry-analysis/antibody-therapy-market, accessed 6 February 2023). Antibody therapy targets diverse molecules ranging from soluble signaling molecules such as cytokines and chemokines, plasma membrane anchored immunomodulatory and growth regulating transmembrane molecules such as CD19, CD20, HER2, and various GPCRs, to viral capsid proteins critical to host cell infection [3,4,5]. The most recent antibody therapy to neutralize the SARS-CoV-2 virus upon infection has brought the term “neutralizing antibody” into the public’s lexicon.

Despite the tremendous success in translational applications, antibodies are complex biologic [1,6] molecules, and have many well-recognized limitations as drugs. The polyclonal antibody purification from an immunized host has given way to a production of a more reliable and consistent quality monoclonal antibody (mAb) through engineering of antibody secreting cell lines [2]. Incorporation of molecular methods to “humanize” antibodies derived from a non-human host has largely resolved the problem of immunogenicity of the antibody. Mammalian host cells (usually CHO cells) transfected with the cDNA encoding the desired antibody assures proper post-translational glycosylation, folding, disulfide bond formation, enabling secretion of the correctly folded antibody. Recent efforts report using yeast and insect cell hosts that share the proper post-translational modifications with mammalian cells, but are easier to achieve greater antibody production [1]. Regardless of the host cell, stringent purification of the antibody limiting the host cell protein to less than one part per million is required for use as a drug [2]. During and after the purification process, the complex biologic antibodies are prone to loss of activity from aggregation, denaturation, fragmentation, deamidation, and oxidation [7].

The success of antibody therapy rests on several unique properties, including the high target specificity, the multimodal mechanisms of antibody recognized-target cell elimination, and the relatively long half-life of days to weeks in circulation, depending on the antibody isotype and subclass [1]. Discovery of antibody replacements that keep the biological activity and desirable properties as a drug, but without the limitations of complex biologic production, and with a superior function, is a major goal [8]. 

Middle-biomolecules (e.g., peptides [6,9], oligonucleotides [10,11], oligosaccharides [12]) which are placed between small synthetic molecules and the large antibodies by their molecular mass, meet the above criteria for potential targeted therapeutics (Table 1). Among the middle-biomolecules, peptides are well developed, with over 60 targeted peptide drugs already approved worldwide [13]. Introduction of artificial structure(s) into a middle-biomolecule, which would often add a superior function to the drug molecule, is easier than that into an antibody. Introducing a reactive warhead structure into such biomolecules to create biomolecular targeted covalent inhibitors (abbreviated as bioTCIs) represents a drug development effort in this direction [6]. TCIs are defined as the subset of covalent drugs that semi-permanently inhibit the target protein activity upon binding [14] and the duration of the drug effect is only limited by the target protein turnover. The covalent binding should be highly specific and the covalent bond only formed with the intended amino acid on the target [15]. bioTCIs which can precisely bind to the target by multi-point molecular recognition satisfy these requirements. 

Here, we review the development of bioTCIs. First, we discuss their history and general considerations of selected “warheads” [16] introduced to the bioTCIs to enable covalent binding to the target receptor. Next, we focus on recent hot topics of middle-molecule-type TCIs, including a concept of a reversible TCIs where the pharmacological action can be easily and specifically reversed by an antidote. Lastly, we review the remaining technical challenges and questions that need to be addressed for a successful future translational application of bioTCI drugs.

## 2. History and General Principle of bioTCI

### 2.1. From Small Molecular TCI to bioTCI

**Figure 1:**
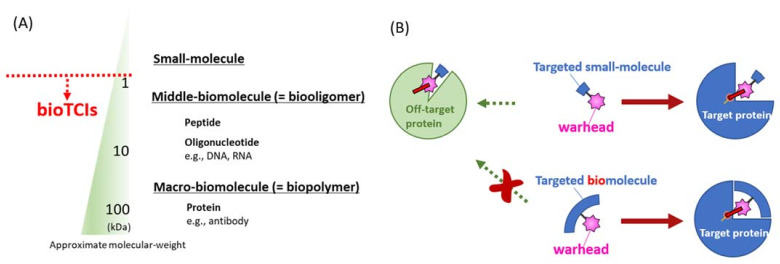
Reported modalities of targeted covalent inhibitors (TCIs). (**A**) Different TCI modalities are categorized as small, middle, or macro-molecules according to their molecular mass. This classification by the molecular mass is not absolute, with many overlaps. Peptidic and nucleotidic aptamers discussed in this review are defined as middle-molecules by such a molecular mass classification. (**B**) The most distinguishing difference between conventional small-molecular TCIs (upper) and bioTCIs (lower). Because of the multi-point recognition of the target protein by the bioTCI, off-target covalent conjugation toward target-unrelated protein will be suppressed. The cost for the stringent target recognition via the molecular-weight increase of bioTCIs is the difficulty in inhibiting intracellular proteins because of the limited membrane permeability.

**Figure 2:**
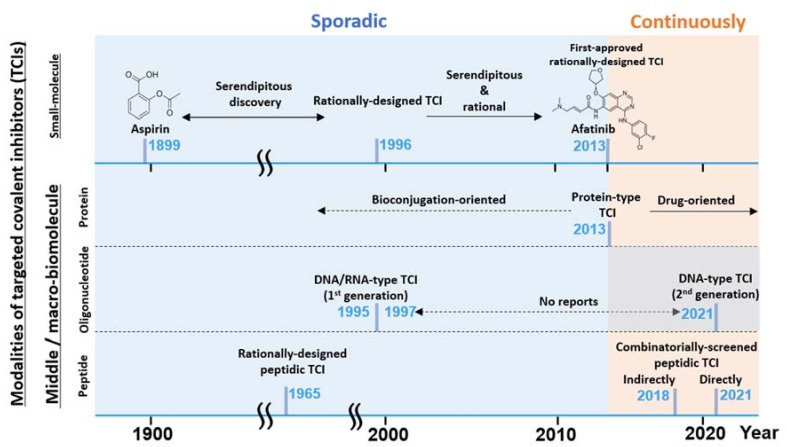
A graphical summary of the brief TCI history and recent hot topics described in this review. TCI modalities listed (left axis) vs. timeline (horizontal axis) and the notable development are listed. TCI development has been mostly sporadic until recently with the renewed interest [15] in TCI drugs.

Figure 1 summarizes the features of the four representative TCI modalities, including the middle-molecules, and Figure 2 shows a simplified history of their development. Among them, small-molecule covalent drugs are not new [17,18,19]. A prototypical example is aspirin, which has been in distribution since 1899, but the covalent mechanism of inhibition was revealed only in the 1970s [20,21]. Indeed, one-third of FDA-approved enzyme inhibitors, including blockbuster drugs such as warfarin, proton-pump inhibitors, and β-lactam antibiotics, of major clinical utility targeting various diseases are covalent drugs [14,15]. Most currently active covalent drugs were discovered by coincidence and the mechanism of covalent inhibition revealed only after the drugs’ usefulness had been established [15]. Rational design of the small-molecule TCI started in the late 20th century [22,23], and the first approved rationally designed TCI appeared in 2013 [14,18]. Thereafter, the numbers of patents, publications, and the FDA approval of TCIs increased dramatically [18,19]. As with conventional drugs, the major development in covalent drugs focused on small molecules because of the desirable features such as easy production, lack of immunogenic response, and the possibility of oral administration. However, it is also well-known that such small molecule covalent drugs, including those that are FDA-approved, show a concentration-dependent off-target binding where the interaction between the small-molecule and a protein target is strongly influenced by the hydrophobic interaction [24,25]. Such hydrophobic interaction domains are ubiquitously present in many proteins, resulting in off-target binding of the small molecules [26]. To circumvent this limitation of small molecule non-specificity, more recent rational TCI design has gradually shifted to TCI bio-oligomers/polymers (i.e., bioTCIs including TCI peptides, proteins, and nucleic acids), which have higher target specificity and selectivity [27,28,29,30,31]. Among the bio-oligomers/polymers, middle molecule-type target binders (i.e., peptides [6] and oligonucleotides [10]) are traditionally recognized as less suitable drug modalities because of the short in vivo half-life from protease and nuclease digestion, and rapid renal clearance. However, enabling these middle molecules to bind covalently to the target protein could prolong the drug action, regardless of the macroscopically observable pharmacokinetic (PK) half-life of the unbound free drug, because of the irreversible nature of covalent binding. Covalent binding to the target protein appears to endow the binders with relative protease [32] and nuclease resistance [33,34], further prolonging the drugs’ in vivo half-life. This shift in the effector molecules from small to middle has resulted in the resurgence of TCI development.

### 2.2. Warhead Design and Introduction into Middle/Macro-Biomolecules

**Figure 3:**
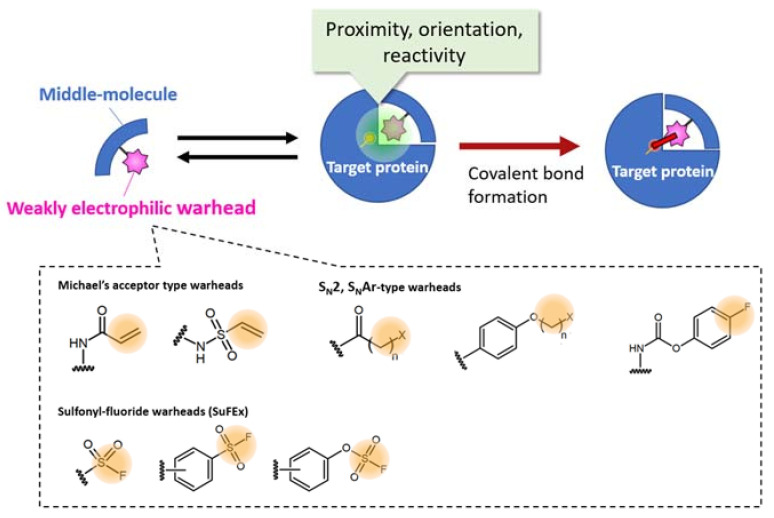
Selected examples of warheads for creating a bioTCI. For the appropriate covalent bond formation, both proximity, orientation, and optimized reactivity between the warhead and a nucleophilic amino acid of the target protein are needed. For a comprehensive classification of the warheads, see [16,40]. Synthetic strategies of SuFEx-type warheads, summarized in the latest review by am Ende and Ball’s group [41] is very informative.

bioTCI combines the inherent enhanced specificity of the middle/macro-biomolecules and covalent-binding to the target protein enabled by incorporating a weakly reactive electrophile (i.e., a warhead) to further enhance the affinity and selectivity for the intended target (Figure 3) [27,28,29]. Many warheads, classified by reactive groups, targeted nucleophilic amino acids, and the mechanism of covalent inhibition, have been reported in the literature and summarized in recent reviews [14,16,35,36]. Choosing the warhead to enable the covalent interaction between the middle/macro-biomolecules and the target is critical in creating a bioTCI. Adjusting warhead electrophilicity is the key for developing covalent drugs [37], regardless of the drug modality; too much electrophilicity would induce non-specific reactions to any nucleophilic functional groups of any unrelated proteins. Thus, weak electrophilic warheads which do not react with the nucleophilic functional group at a dilute concentration are needed [38,39]. For example, Michael-addition, conventional nucleophilic substitution-(S_N_2, S_N_Ar), or sulfur fluoride exchange-(SuFEx) reaction-type warheads have been often used in the development of bioTCIs (Figure 3) [29].

**Figure 4:**
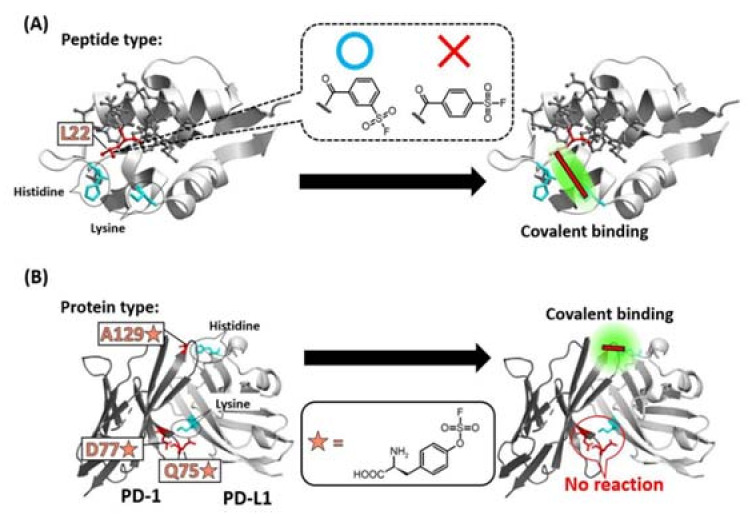
Specificity of the position of warhead incorporation for a successful covalent-bond formation. (**A**) SuFEx-type warhead isomers were introduced into the 22nd leucine (L22) position of a Mdm2/4 (light gray) binding staple peptide (dark gray). Only meta-substituted regioisomer reacted with a lysine of Mdm2/4. (**B**) Q75, D77, A129 of PD-1 (dark gray) was mutated into SuFEx-type Uaa (orange star). Only A129-mutated PD-1 reacted with a histidine on PD-L1 (light gray).

In most cases, bioTCIs are created by a rational design through introduction of the warhead into the targeted middle/macro-biomolecules at a specific position (i.e., placement) [29] via chemical modification [31] including bio-conjugation [28,42] or unnatural amino acid (Uaa) incorporation via genetic code expansion [30]. Usually, the position is determined based on the three-dimensional structure of an inhibitor/target complex. The rationale is that by introducing a warhead to an appropriate position of the binder, the location of the warhead becomes physically closer to a destination nucleophilic amino acid of the target protein. This proximity effect increases the local effective concentration of the warhead/nucleophilic amino acid and promotes the appropriate covalent binding reaction [15,18,29,38,39]. The positional determination is often time-consuming, and the covalent bond sometimes cannot form despite the seemingly appropriate introduction a warhead to the inhibitor. In fact, when a SuFEx-type latent-electrophilic warhead [16,41,43,44,45], which can theoretically react with any nucleophilic amino acids [46,47,48], was introduced into the 22nd leucine (L22) position of a Mdm2/4-binding staple peptide guided by the structural information, a covalent bond did not form despite a histidine and lysine in the warhead’s vicinity on the target protein. Unpredictably, the expected covalent bond formed when a regioisomer of the warhead was used (Figure 4A) [49]. When a SuFEx-type Uaa-warhead was rationally introduced into the 75th glutamine, 77th asparagine, and 129th alanine (Q75, D77, A129) of the PD-1 protein, respectively, only A129-mutated PD-1 reacted with PD-L1 (Figure 4B) [50]. These results suggested that a simple proximity between the warhead and the receptive target amino acid may not be sufficient to facilitate the covalent reaction; stringent proximity and proper orientation, as is typical for a S_N_2-type reaction, between the warhead and the nucleophilic amino acid may be needed. A deeper understanding of the “matchmaking environment” [38,39,46,51] surrounding the warhead enabling the covalent reaction with the target is desirable. Rationale approaches based on structural information have not consistently worked and a more robust alternative method, not simply relying on trial-and-error for optimal warhead placement, is essential for this important area of drug development to progress.

Among the bioTCIs, the development of peptidic inhibitors started early (i.e., in the middle of 1960s) [52] because position-specific chemical modification of the warhead can be accomplished through the historically established solid-phase peptide synthesis [53] followed by the post-synthesis chemical modification [31]. Besides the rational introduction of a warhead into the targeted peptide, both irreversible- and reversible-peptidic TCIs have been discovered, starting from screening of natural products [54]. The former and the latter examples are peptide-epoxides (e.g., epoxomicin) [55], and peptide-aldehydes (e.g., flavopeptin) [56], respectively. Structurally optimized variants of the natural peptides (i.e., peptidomimetics [57,58] TCIs) have been an active area of investigation. Natural peptides and peptidomimetics are both included as peptidic TCIs in this review. Indeed, the peptidic TCIs are the most developed and promising modality among the bioTCIs, and currently several (e.g., Carfilzomib) have been approved by the FDA [59]. Current progress and modern history of the peptidic TCIs are summarized in informative recent reviews [27,29].

In contrast, the development of the proteinic TCIs has been slow because the specific chemical modification of a protein is a huge challenge and, traditionally, only the Uaa incorporation methodology [60] has been performed. However, the warhead-endowed Uaa promiscuously reacts to off-target biomolecules resulting in interruption of translation or cytotoxicity [29]. This promiscuous reaction has been elegantly overcome by Wang in 2013 through fine-tuning of the Uaa-warhead electrophilicity by proximity-enabled reactivity such that the Uaa does not react with off-target natural amino acids and other biomolecules under physiological conditions [50]. With this breakthrough, the proteinic TCI development has sped up [61,62] as well-summarized in recent reviews [29,63].

### 2.3. Pros and Cons of bioTCI over Non-Covalent Biomolecular Targeted Inhibitors

**Figure 5:**
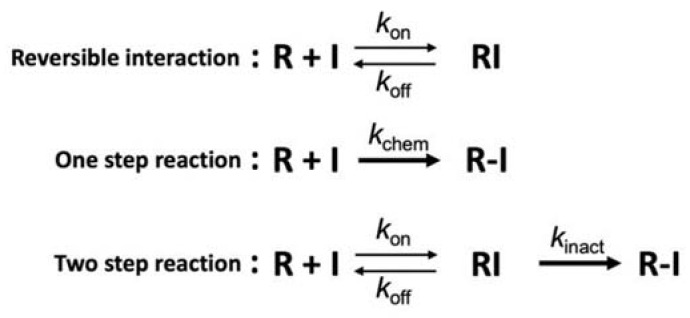
Mechanisms of covalent interaction [65,66]. Interaction mechanism between the protein (R) and the inhibitor (I) of non-covalent-type (**top**), conventional covalent-type including non-specific conjugation (middle) or targeted covalent-type (**bottom**). *k*_inact_ and *k*_chem_ are defined as the inactivation rate constant for the 2-step irreversible inhibition and the reaction rate constant for the 1-step irreversible conjugation, respectively. For the TCI (bottom), first step equilibrium (*k*_off_/*k*_on_) is a part of the inactivation constant (*K*_I_ = [*k*_off_ + *k*_inact_]/*k*_on_), which includes the TCI’s *affinity* and resembles the concept of the dissociation constant of a non-covalent binder (i.e., *K*_d_ = *k*_off_/*k*_on_; top). However, overall performance of the TCI is represented as a *non*-equilibrated parameter of *k*_inact_/*K*_I_ (i.e., the inactivation efficiency).

A major advantage of the bioTCI not shared by non-covalent biomolecular drugs is the prolonged duration of the drug effect and a less frequently required dosing, lessening the burden to patients. As shown in Figure 5, kinetic studies of the covalent bond formation between the TCI and the target protein follow a two-step process where the reversible initial docking of the compound to the target is followed by an irreversible covalent bond formation, resulting in a drug–protein conjugate that is not affected by the classical equilibrium kinetics of binding. Instead, the overall inhibition efficiency of TCI is better described by a derived PK parameter *k*_inact_/*K*_I_ accounting for the irreversible second step binding [14,18,38,39,64,65,66]. A corollary to this two-step target recognition is that a non-specific covalent bonding of the warhead to the target seldom occurs, which is desirable and a consequence of the choice of warhead with reduced reactivity but increased specificity. The compromise between reactivity and specificity also results in a rather slow covalent bond formation requiring 10s of minutes to hours for completion. However, this alone is not a major drawback for a practical application since drugs are often continuously infused to attain a specific clinical endpoint and the bioTCI could be continuously infused to maintain the required serum concentration of the drug for the duration necessary for covalent bond formation with the target. The irreversible nature of the covalent bond formation assures us that, even if the bioTCI shows a relatively low affinity, the gradual shift in the equilibrium between the free and drug-bound target should cause a complete inhibition of the target. The non-equilibrium covalent binding of the bioTCI would also overcome any competing endogenous substrate(s) which binds to the same docking site of the target protein [18]. A prolonged inhibition of the target protein is expected from the extension of the pharmacological half-life regardless of the half-life of the free drug. The drug effect of the covalently bound bioTCI should far outlast even after the clearance of the unbound drug in the serum. An excellent theoretical treatment of TCI kinetics can be found in [64], and experimental data for small molecule TCI [67] and aptamer TCI [33,34,68] have been reported.

Given the long drug effect, the potential risk for irreversible adverse drug effects (ADE) by TCI binding to off-targets has been a major concern, and perhaps the main reason for the hesitancy for a wide acceptance of TCI as a drug platform. To minimize ADEs, TCIs require exquisitely high target specificity [15]. Although many small molecule-, peptide-, and protein-type covalent drugs have been developed, none have overcome the potential risks of irreversible ADEs. In the following section, we discuss a very recent development in reversible peptidic and nucleotidic TCI where, in the latter, the pharmacological effect is reversible even while still covalently bound to the target.

## 3. Recent Hot Topics of bioTci

### 3.1. Combinatorial Screening of Peptidic TCI: A Well-Developed Modality

**Figure 6:**
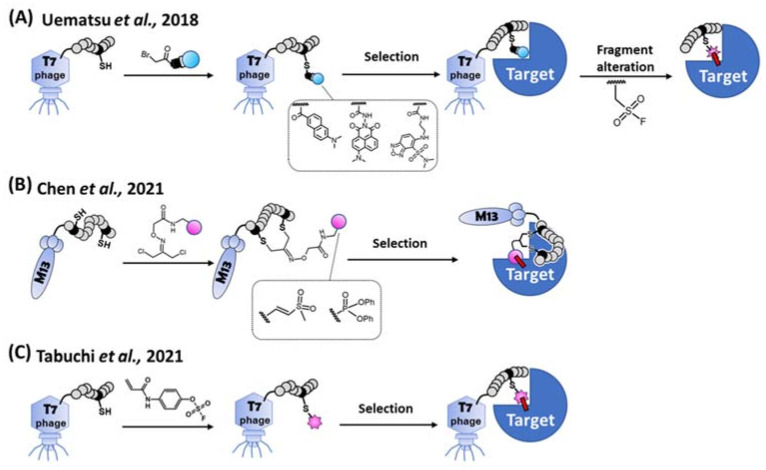
Methods for the combinatorial screening of peptidic covalent binders. (**A**) Indirect screening. Library peptide on the T7 phage is modified by each bait fragment (i.e., unreactive mock warhead), respectively. After the selection of a target protein, a consensus sequence of a peptide is obtained. The bait fragment of the peptide is alternated to a SuFEx-type warhead for obtaining a covalent binder [70]. (**B**) Bogyo’s direct screening method. A cysteine-reactive vinyl sulfone or a serine-reactive diphenylphosphonate is introduced to the library peptide on the M13 phage. A covalent binder is directly selected from the warhead modified cyclic peptide library [71]. (**C**) Our direct screening method. Aryl-fluorosulfate (AFS) warhead is introduced to the library peptide on the T7 phage. A covalent binder is directly selected from the AFS-modified peptide library via reactivity and affinity-based co-selection [51].

Despite the long track record of success, rational design and/or natural screening for peptidic TCIs cannot meet the demands of the ever-increasing broad range of different target proteins. Alternatively, combinatorial screening methods are widely used to discover peptidic binders as they allow for the rapid generation of a candidate library with a large diversity [69]. Theoretically, peptidic TCIs [27,29] can also be obtained via the combinatorial screening by introducing a warhead into a designated position of the library peptides. Practically, control of the warhead reactivity [40] during the library construction and selection is difficult, and the warhead in the library often forms promiscuous covalent bonds between biomolecules [70]. To get around this problem, an *indirect* combinatorial method was implemented to first select for a targeting peptide using a mock-warhead-introduced peptide library on the T7 phage. After the selection and peptide-sequence identification, the desired target-selective covalent binding was observed when the unreactive mock warhead was replaced by a reactive warhead (Figure 6A) [70].

In 2021, *direct* combinatorial screening via the phage display was independently reported by Bogyo’s group and us (Figure 6B,C). Bogyo’s group designed two least-reactive warheads to minimize the promiscuous reactions between the biomolecules, and successfully selected peptidic TCIs using the M13 phage display [71]. They stringently regulated the reactivity of each warhead against cysteine or serine independently, and a bifunctional linker attached to each warhead. The free ends of the bifunctional linker were conjugated with two designated cysteines on a randomized peptide via the thioether linkage. After bio-panning using the warhead-introduced peptide library, cyclic peptidic TCIs against cysteine- and serine-proteases were obtained. Using another approach, Taki’s group introduced a latent-electrophilic aryl fluorosulfate (i.e., fosylate [72]; Ar-OSO_2_F) warhead [41,73] which is completely inert and activated only in a matchmaking (i.e., enzyme-like) microenvironment [38,39,46] created between the target protein and an appropriate peptide during the reactivity and affinity-based [74] co-selection process of the T7 phage display [51]. The fosylate warhead minimized the promiscuous reaction during the library’s construction/selection, and a TCI was obtained with only 2 rounds of bio-panning. Non-specific and non-covalent interactions between target-unrelated biomolecules were eliminated during a harsh washing step with a urea and SDS containing buffer, while the robust T7 phage still kept its infectivity [75].

Another direct screening system using M13 phage display extended the possibility of finding reversible peptidic TCIs, as demonstrated by Zheng and Gao [76]. A lysine-targeted warhead (i.e., 2-acetylphenylboronic acid) was attached to a library peptide displayed on the phage, and cyclic TCIs reversibly conjugating to SrtA and SARS-CoV-2 spike RBD proteins were selected. This new modality endowed a long target-residence time of the drug without permanent conjugation, as in bioTCIs, and reduced the drug clearance and risk of immunogenicity [18,27]. A reversible covalent binding of cyclic TCIs to off-targets should reduce the chances of a prolonged ADE [77].

Antidote-reversible small-molecule TCIs targeting thiols, alcohol, and amines have been described [78], but none have been reported for a peptidic TCI. These approaches may be better described as a reversal of the covalent bond formation rather than an antidote reversal of the drug effect, even while the TCI is still covalently bound to the target protein. A truly neutralizable warhead/antidote pair (e.g., benzoxaborole/reduced-glutathione) [79,80], where the covalent bond is maintained but the non-toxic antidote rendering the drug inactive, has been proposed for peptidic TCI, but this technology is yet to be implemented. Discovery of a technology that will allow on-demand and specific reversal of peptidic TCIs by addition of non-toxic antidote molecules, as discussed in the next section for nucleotide TCIs, is one key future direction.

### 3.2. Nucleotidic TCI: A Developing Modality

Single-stranded DNA or RNA oligonucleotides form complex folded structures and a specific aptamer that binds to the desired target [81] can be identified by a repetitive screening of an aptamer library against the target by a process termed systematic evolution of ligands by exponential enrichment (SELEX) [82,83,84]. The starting library of N random sequence possesses 4^N^ complexity (i.e., ~10^12^ for N = 20, and 10^24^ for N = 40), favoring the discovery of an aptamer with a specific sequence necessary for binding to the desired target. Many high affinity aptamers with an affinity in the pM range or better have been selected, curated (Apta-Index, http://www.aptagen.com/; RNAapt3D, https://rnaapt3d.medals.jp/, both accessed on 6 February 2023) and readily available from many commercial vendors. Aptamers have been touted as potential antibody replacements given their high specificity and affinity [83,85] but the limitation of a very short in vivo half-life has prevented their practical application [81]. In theory, these already identified aptamers can be rendered potential TCIs with a long drug half-life just by incorporating a warhead, as recently demonstrated for the thrombin binding aptamer [28,34] and SARS-CoV-2 spike protein binding aptamer [42,68]. Whether other warhead-introduced aptamers will conjugate with the target protein depends on the orientation of the aptamer to the target dictating the proper positioning of the warhead on the aptamer, and the availability of the interaction-capable amino acids on the target protein. Such a trial-and-error approach to creating a TCI from a pre-selected aptamer can be circumvented by directly selecting for a covalently binding aptamer during the SELEX process akin to the phage-library approach established for the peptidic TCIs.

Smith and colleagues were the first to report covalently binding RNA and DNA aptamers targeting the neutrophil elastase [86,87] obtained by a direct combinatorial screening method denoted the blended SELEX (Table 2). This first-generation nucleotidic bioTCI was selected by utilizing a splint-DNA comprising a small-molecule-TCI as the elastase-specific warhead, a spacer, and a 3′ fifteen base pair overlap complimentary to the forward primer region of the aptamer library. The mobility-shifted aptamer-bound protein band was gel-isolated, and the eluted aptamer was amplified by PCR to generate the aptamer pool for the second-round selection. The optimized bioTCI from the library enhanced the target selectivity and specificity of the original small-molecule-TCI. This method has a major advantage in that an unmodified conventional aptamer library is used as the input. The selected aptamer can be readily separated from the covalently bound splint DNA by heating, since only the partial double strand connects the splint DNA to the unmodified library aptamer pool. An expansion of this attractive method of combinatorial screening for nucleotidic bioTCIs using a *generalized* warhead capable of interacting with any desired target protein, instead of the neutrophil elastase-specific already-known TCI as the warhead, has not been reported.

**Figure 7:**
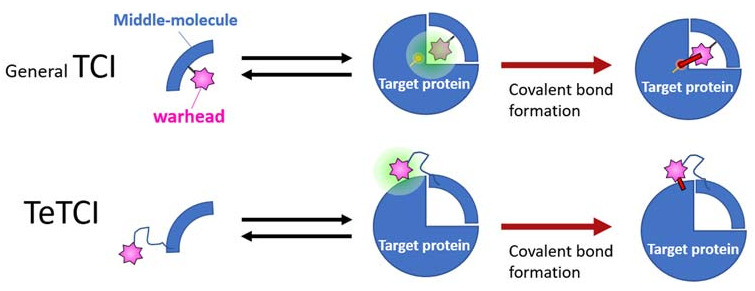
A graphical summary of the conventional vs. tethered TCI (abbreviated as TeTCI). The general TCI (top row, blue quarter) endowed with a warhead (pink star) follows a two-step binding to form a covalent bond with the amino acid (s) usually within the TCI docking domain of the target protein (right). A TeTCI (second row) where the warhead attaches to the drug modality (e.g., a nucleotidic aptamer) through a long linker similarly binds to the target, but the site of covalent attachment is outside the docking domain. This distinction between the drug docking site and the site of covalent bond formation (i.e., general vs. TeTCI) is not modality dependent since some small molecule TCI endowed with a warhead with a long linker also reacts with residues outside of the presumptive docking domain.

**Figure 8:**
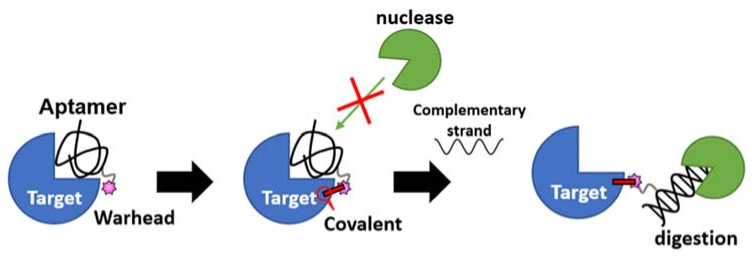
A reversible antidote mechanism of nucleotidic TCI. Once the irreversible covalent bond is formed on the target, the aptamer becomes nuclease (green packman) resistance. When the aptamer is dislodged from the binding site by the on-demand addition of the complimentary strand antidote, it becomes sensitive to nuclease digestion even though still covalently bound to the target.

The second-generation nucleotidic bioTCI is based on a pre-identified DNA aptamer showing high affinity for the target, and a warhead directly conjugated at a specific position of the DNA (Table 2) [28,34,68]. It should be noted that TCI aptamers created in such a fashion are better described as a tethered-TCI (TeTCI) (Figure 7) since the protein domain forming the covalent bond is outside [14,88,89] the actual aptamer docking domain. The long linker between the warhead and the aptamer serves as a chain that tethers the aptamer to the target protein. The TeTCI is conceptually different from most of the small molecule or even the peptidic TCI where the covalently binding residue is within or near the docking domain [15]. Three methods for introducing a warhead into an aptamer to create a TeTCI have been reported over the last two years. Tabuchi et al. [28] replaced a thymine (T) residue of the thrombin binding aptamer (TBA) with an octadiynyl-dU (OctdU) containing an alkyne group based on the structural information of TBA-bound thrombin [90], and a benzenesulfonyl-fluoride warhead introduced by the copper-catalyzed azide–alkyne cycloaddition (CuAAC; also known as a click chemistry). The starting 15-mer TBA aptamer selected by the conventional SELEX is a well-studied thrombin binding G-quadruplex aptamer [91]. Warhead introduction at the 3rd T residue of the TBA (i.e., TBA3) resulted in an efficient TCI reacting with thrombin, while the same warhead placed at T9 facing away from the target protein resulted in only a weak covalent-binding ability (Figure S1). The resulting TBA3 covalently bound to thrombin and inhibited the enzymatic activity of the target. TBA3 demonstrated nuclease resistance, and the TeTCI remained bound to thrombin, and intact, even after 24 h of digestion with DNase I [33]. As expected, the addition of the complimentary-strand (CS) oligonucleotide against the aptamer sequence as an antidote [92] reversed the thrombin inhibition, and the CS antidote rendered the thrombin-conjugated TBA3 nuclease-sensitive [28]. The reversal by the CS antidote was swift, probably because the relatively long tether placed between the warhead and the aptamer did not interfere with either the double-strand (DS) formation between the aptamer and the CS, or the exposure of the DS towards the outside of the binding pocket on the target protein accessible to nuclease digestion (Figure 8). We have applied the same technology and confirmed the creation of a TeTCI targeting SARS-CoV-2 S-protein RBD domain (Figure S2) from a previously reported DNA aptamer. Multiple warhead introduction into a single aptamer showed greater inhibition than the corresponding monoadduct.

TBA possessing an alternative inverse electrophile, as reported by Tivon et al., showed the same results. The conjugation efficiency of their aptamer TCI with thrombin depended on where in the aptamer the warhead was introduced, and the enzymatic inhibition and relative nuclease resistance of the aptamer-conjugated thrombin were reversed by the CS antidote [34]. The availability of precise structural information of TBA bound to thrombin enabled the rational determination of where to introduce the warhead for this aptamer. Structural information is usually unavailable for most aptamers bound to its target, and the determination of the position of warhead introduction becomes a labor-intensive trial-and-error process where every T residue is replaced with an OctdU and screened for efficient covalent binding to the target. Qin et al. [68] reported a potential docking-structure-independent method of warhead introduction by simply extending the 3′ end of an aptamer with a phosphorothioate (PS)-linked nucleic acid tail, and subsequent introduction of a warhead through a simple nucleophilic reaction between a Br-warhead and the S-atom of the PS linker. The authors chose two SARS-CoV-2 S-protein binding aptamers previously selected by the conventional SELEX [85,93] and tailed the 3′ end with 7 T residues possessing 1, 3, 5, or 7 PS bonds. Subsequent methyl-benzenesulfonyl fluoride introduction by the nucleophilic substitution reaction rendered the aptamer a TCI with the 7 PS-tail showing the best covalent bond formation with the target protein. Whether this structural information-independent approach to create a TCI by the PS tailing can be extended to other aptamers or whether the PS-tailing bound aptamer is reversible by the CS antidote is unknown.

TeTCI, created by introducing a warhead with a relatively long linker, might increase the non-specific binding to unintended off-targets. TeTCI examined to date show specificity for the intended target in the presence of serum, suggesting that even a tethered warhead requires a proper matchmaking environment guided by the aptamer docking to the intended target for the covalent bond formation. However, further studies are needed to determine whether the TeTCI’s matchmaking environment is as selective and rigorous as the conventional TCI where the docking itself appears to create the environment conducive to the covalent bond formation. Alternatively, incorporation of sulfamoylfluoride-functionalized nucleosides [94] where the modified nucleosides can directly undergo SuFEx with the target with no long linker could result in a traditional nucleotidic TCI where the site of covalent bond formation is within the docking pocket.

## 4. Future Perspectives of bioTCI Research: Technical Challenges and Critical Questions That Need to Be Answered

For the re-surging interest in bioTCI to transform into a true next-generation antibody replacement platform, many obstacles remain. Here, we discuss some of the outstanding technical and conceptual issues.

### 4.1. Design and Selection Methodology of bioTCI

A major challenge in developing bioTCI is determining the most appropriate position(s) of the warhead introduction. When available, the optimal position is determined based on the three-dimensional structure of the inhibitor/target complex. As discussed above, the docking structural information is most often not available and ultimately such a rationally designed bioTCI may not form a covalent bond with the intended target. TCI, by definition, must have a negligible binding to unintended off-target while exhibiting high affinity for the intended target. The warhead must exhibit a profound specificity when introduced as a bioTCI when a precise “matchmaking” microenvironment [38,39,46,51] is established. The precise nature of the matchmaking microenvironment is currently unknown, but a simple focused introduction of a warhead into a non-covalently binding precursor drug may not facilitate the intended covalent reaction; stringent proximity and orientation between the warhead and the nucleophilic amino acid are needed. Currently, the methods addressing such problems are limited, although computational approaches for simulating the detailed interaction between a warhead and the target residue could shed some light [95,96].

An alternative approach for identifying a bioTCI is the combinatorial screening for selecting for a covalent binder rather than modifying a pre-existing non-covalently binding biomolecule. Such a combinatorial TCI selection for peptides, especially using the phage-display platform, is better developed, as discussed above in the peptidic TCI section. There is no report of a generalized combinatorial screening for a direct screening for covalently binding nucleotidic aptamer despite the early reports of selecting for the first-generation nucleotidic TCI through the blended SELEX. Generalization of the blended SELEX approach where an electrophilic warhead-conjugated splint-DNA forms a partial double strand with the forward primer region of the aptamer library should allow a direct combinatorial selection of a covalently binding aptamer. This approach of direct selection for a third generation nucleotidic TCI approach has an advantage in that the unmodified aptamer library used for a conventional SELEX can be used with only the addition of a warhead endowed splint-DNA required. Covalent binding between the target and the splint-DNA would occur outside of the library-aptamer binding site, so subsequent PCR amplification of the bound-aptamer on the target should not be a problem. In contrast, a direct warhead conjugation on the 3′ end of a library-aptamer molecule, for example, by a 3′ PS-tail extension, and covalent binding of the aptamer to the target protein will require a complete protease digestion for the SELEX amplification process. The target-bound aptamer should possess only a single (or a few) amino acids derived from the target protein for the selected pool to act as the template for an efficient PCR amplification. Alternatively, incorporation of a modified nucleotide triphosphate such as 5-Ethynyl-2′-deoxyuridine-5′-triphosphate (EdUTP) during the PCR amplification step and a subsequent bioconjugation of a warhead to regenerate a warhead-endowed library (i.e., click-SELEX [97]) should allow repetitive rounds of selection and amplification, but this approach will select for an aptamer with multiple warheads (e.g., all T residues replaced with a warhead-endowed EdU). Whether the actual docking of the aptamer to the target is adversely affected by multiple warheads and whether such a TCI will remain reversible by the CS antidote is unknown.

### 4.2. Beyond the Target Affinity/Specificity: Additional Functionalization of bioTCI

**Figure 9:**
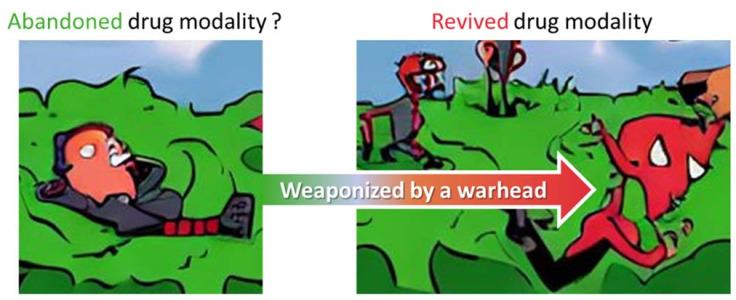
Weaponized and revived aptamer: From zero to hero. A conceptual summary of how the abundance of aptamers reported in the literature, but currently abandoned from clinical translation, could be transformed into potentially useful drugs by warhead introduction, rendering them to tethered-TCIs.

A conceptual limitation of a bioTCI as an antibody replacement originates from the fact that antibodies serve two major functions of target specificity and effector trigger mediated by the Fab and Fc portions of the antibody, respectively. bioTCI can mimic the specificity and high affinity for a target likely replicating the target neutralizing ability of an antibody. bioTCI should inhibit infection of cells by pathogens and disrupt various signaling by inhibiting protein–protein interactions (PPIs) between extracellular ligands and receptors. For example, DNA aptamers targeting the SARS-CoV-2 spike protein have been reported to inhibit its binding to the angiotensin-converting enzyme 2 receptor necessary for viral particle infection of cells [93,98]. Although not tested in vivo, a recently reported covalently binding anti-spike-protein nucleotidic TCI [68] should be effective as a long-lasting antibody equivalent neutralizing viral infection. Several aptamers targeting the check point proteins and their ligands have been reported, and a similar warhead introduction into these aptamers could create bioTCIs disrupting the immune check point mechanism used by cancer cells to evade elimination by the immune system. Such TCIs, much smaller than antibodies, may enable better infiltration of solid cancers reported to be less responsive to mAb checkpoint inhibitor therapy [99]. Aside from the checkpoint proteins, many therapeutically critical molecules have been the target for mAb therapies and aptamers have been reported for many of these targets (Table 3). These aptamers are not seeing immediate translational applications because they are prone to nuclease digestion and rapid renal clearance [84], in vivo, but could be transformed into clinically useful drugs as bioTCIs. We believe that a simple warhead introduction into many of the already-known but clinically abandoned aptamers could revive them as useful agents for translational research (Figure 9). Further engineering of bioTCIs to enable dual-capture of the antigen mimicking the two Fab arms of the antibody may even recapitulate the antibody-mediated cross-linking of antigen and bind to circulating components of the complement pathway leading to passive protection and complement-dependent cytotoxicity. Dual-capture bioTCIs could show increased potency and decrease the development of cellular resistance from inadequate dosing and limited targeting, as has been shown for bi-specific antibodies [100].

The lack of Fc analogue in bioTCI could limit the cell-mediated cytotoxicity effector function. The antibody Fc, as a part of the immune complex decorating the target cell, binds to the membrane-bound Fc receptor (FcR, e.g., FcgRIIIa in the case of NK cells) of effector cells, triggering their cytotoxic activity [161]. A dual-pronged effector mechanism of cytotoxic granules release containing perforin and granzymes, and induction of death receptor-mediated apoptosis by expressing TRAIL and/or Fas ligand (FasL) to engage TRAIL-R1/-R2 or CD95/Fas, respectively, on the surface of diseased cells are essential for many immunotherapies against cancer [162]. Many details of the interaction between Fc and FcR at the immunological synapse are still unknown, but the Fc binding induces FcR aggregation, phosphorylation of the cytosolic domain of the FcR, and microtubule polarization [163]. While a dual-specific bioTCI that incorporates a FcR-binding motif in addition to the antigen binding motif, in theory, could be created, whether the ligation of the FcR by a non-Fc peptidic or nucleotidic bioTCI will trigger the effector mechanism is unknown since triggering of the effector signaling seems to require a precise geometrical orientation between the ligand and FcR [163]. However, a TCI targeting the FcR itself inhibiting antibody binding is in line with the trend of developing novel drugs for autoimmune diseases [164]. One promising report describes an efficient and highly specific bioconjugation of the expressed Fc fragment with bio-oligomers (i.e., peptides and nucleotides) using the strain-promoted azide-alkyne cycloaddition reaction [165]. The same technology applied to the bioTCIs resulting in a bioTCI-Fc hybrid, with the target specificity provided by the TCI and the cellular effector provided by the Fc, might recapitulate both functions provided by an antibody.

### 4.3. Other Possibilities

Despite the above potential limitations of TCIs, many of the antibody enhancements leading to more efficacious drugs can apply to TCIs as well. TCIs can serve as a scaffold for conjugating therapeutic molecules such as cytotoxic or radiotherapeutic agents akin to antibody–drug conjugates [166]. TCIs could decorate the surface of liposomes or nanoparticles for enhanced targeted delivery of the cargo [167]. The inherent property of both peptidic and aptameric TCIs enabling synthesis without requiring a biological production host already distinguishes these drugs from conventional antibodies and other biologics. TCI potentially maintaining activity after internalization [154,168] by cells opens the intracellular molecules as therapeutic targets, and is a property not shared by antibodies. Middle-molecular TCIs suitable for inhibiting internal smooth/flat protein surfaces typically responsible for PPIs would be excellent drug candidates for targeting intracellular signaling molecules. Alternative mAb delivery by inhalation [169], enhanced cellular cytotoxicity through alteration of post-translational modification [170]^,^ and activatable antibodies triggered by proteases, extracellular ATP, or a mildly acidic environment [171], to name a few, are ongoing. These technological and conceptual advancements in antibody therapeutics could be incorporated into better bioTCI development as well.

In the long run, the synthesis of both peptides and oligonucleotides should move away from the currently prevalent legacy technologies producing toxic waste products, to a green-synthesis platform [172,173] transforming bioTCIs into true drugs of the future. Exploitation of an automated enzymatic synthesis of oligosaccharides [174], a hitherto undeveloped modality of the middle-biomolecule, may open the door for both combinatorial screening and eco-friendly bioTCI production.

## 5. Conclusions

Monoclonal antibody therapy has had a great impact on many areas of medicine, but most significantly in treating cancer patients, and the refinement of antibody to make it a better drug is likely to continue. A search for an alternative drug platform mitigating the limitations of an antibody is speeding up. bioTCI, because of its high specificity and affinity for the desired target, has been suggested as an antibody alternative. However, TCI has not gained widespread acceptance as a drug platform because of the potential for a prolonged and irreversible side effect. bioTCIs can stringently bind to target proteins because of the multi-point molecular recognition and ameliorate the drawback of small molecule TCIs. A reversible bioTCI, especially an aptamer TCI, where the drug effect can be reversed on demand by a selective CS antidote, should mostly mitigate the major concern of a long-lasting side effect from an irreversible covalent bonding. In addition, bioTCIs should show prolonged in vivo half-life, much like mAbs, from covalent binding to the target protein and nuclease/protease resistance. We believe these advantages provided by the bioTCI can overcome the major obstacles to the therapeutic application of middle biomolecules and speed up its translation to clinical applications as antibody alternatives.

## Figures and Tables

**Table 1:** A qualitative comparison of properties between antibody and middle-biomolecules as targeted drugs. The pros and cons between mAb, peptide, and nucleotide are described. Significant distinguishing features are noted in bold. In vivo half-life of the middle-biomolecules would be improved by attaching warheads to create bioTCIs; details are described in Section 3.

Properties	Antibody	Peptide	Nucleotide
Molecular mass	~150 kDa for IgG	variable (~110Da/residue)	variable (~340Da/residue)
Production	complex biologic	**Synthetic**	**Synthetic**
Mechanisms of target elimination	**multiple**	neutralization	neutralization
Combinatorial screening	limited	**yes**	limited
In vivo half-life	**long**	Short	extremely short
Reversibility	no	yes	**yes (on-demand)**
Immunogenicity	yes	depends	**low**

**Table 2:** A comparison of currently reported nucleotidic TCI. One first generation (DNA and RNA aptamers) original, and three second generation nucleotidic TCIs have been reported in the literature over 2021–2022. Some of the key differences are listed.

Generation	Lead Pharmacophore	Obtained Function *by Lead Engineering*	Warhead Incorporated	Breakthrough Point
1st: Charlton, 1997	Low molecule; TCI	Increasing affinity/specificity *by DNA library conjugation*	valyl phosphonate	- Establishment of combinatorial selection system (i.e., blended SELEX)
2nd: Tabuchi, 2021	Middle molecule; non-covalent DNA aptamer	Nuclease resistance by targeted covalent binding via warhead introduction	4-(acetyl)-benzene-1- sulfonyl fluoride	- Optimization of warhead structure/position - On-demand reversal of semipermanent drug action by a CS antidote
2nd: Tivon (2021)	“	*“*	acyl-sulfonamide	- Warhead introduction by inverse nucleophilic reaction - On-demand reversal
2nd: Qin (2021)	“	*“*	4-(methyl)-benzene-1- sulfonyl fluoride	- Warhead introduction by 3′PS oligo extension - Reversibility by CS antidote not explored

**Table 3:** Aptamers against common immunomodulatory molecules targeted by mAbs. The table lists some of the major molecular targets of mAbs currently FDA approved or in late-stage clinical trials (denoted by *). Aptamers reported interacting with the same targets are listed. The list is not comprehensive and only representative candidates are listed where several aptamers have been reported. The list excludes aptamers, even those in clinical trials, where there are no reports of FDA approved mAbs sharing the same target. Excellent summaries of aptamers in clinical trials can be found in [101,102,103]. Information from several excellent reviews [1,10,104,105,106] and an original literature search was used to gather the information shown here.

Molecular Target	Name		mAb	Aptamer	Reference
**Check point proteins**					
4-1BB	TNF ligand superfamily member 9		Utomilumab *	M12-23	*[107]*
B7-H3	B7 homolog 3		Enoblituzumab *		*none*
BLTA	B and T lymphocyte attenuator		Icatolimab *		*none*
CTLA-4	cytotoxic T lymphocyte-associate antigen 4		Ipilimumab, Tremelimumab	aptCTLA-4	[*108,109*]
ICOS	inducible costimulatory		Vopratelimab *	MRP1-ICOS	[*110*]
Lag-3	lymphocyte-activation gene 3		Relatimab	Apt1	[*111*]
PD1	programmed cell death protein 1		Cemiplimab, Dostarlimab, Nivolumab	MP7	[*112,113,114,115*]
PD-L1	programmed death ligand 1		Atezolizumab, Avelumab, Durvalumab	aptPD-L1	[*116,117,118,119,120,121,122,123,124*]
TIGIT	T cell immunoreceptor with IgG and ITIM domains		Tirabolumab *		*none*
TIM-3	T cell immunoglobulin mucin 3		Sabatolimab *, Cobolimab *	TIJM3Apt	[*125,126*]
**Cytokines/Chemokine**					
BAFF	B-cell activating factor		Belimumab	R1-14	[*127*]
IL1b	interleukin 1 beta		Canakinumab	AptIL-1b, many	[*128*]
IL2	interleukin 2		Basiliximab, Reslizumab	M20 (@mouse)	[*129*]
IL5	interleukin 5		Mepolizumab, Reslizumab	19 clones in 5 families	[*130*]
IL6	interleukin 6		Tocilizumab	S1025, S1026, AIR-3	[*131,132,133*]
IL12	interleukin 12		Ustekinumab		*none*
IL-13	interleukin 13		Tralokinumab		*none*
IL17a	interleukin 17a		Ixekizumab, Secukinumab	Apt21-2	[*134*]
IL23	interleukin 23		Guselkumab, Tildrakizumab	clone 1, clone A5, C3	[*135,136,137*]
IL36	interleukin		Spesolimab		*none*
TNFa	tumor necrosis factor alpha		Adalimumab, Certolizumab, Golimumab	AptTNF-a, VR11	[*128,138*]
**Tumor markers**					
CD3	cluster of differentiation	3	Teplizumab *	J7, OSJ-T1-4	[*139*]
CD4	“	4	Ibalizumab	U26	[*140*]
CD19	“	19	Tafasitamab, Blinatumomab	B83.T2	[*141*]
CD20	“	20	Rituximab, Ibritumomab, Obinutuzumab	AP1-3	[*142*]
CD22	“	22	Inotuzumab, Moxetunomab, epcortamab *		*none*
CD30	“	30	Brentuximab	C2, NGS6.0	[*143*]
CD33	“	33	Gemtuzumab	S30	[*144*]
CD38	“	38	Daratumumab, Isatuximab	aptamer#1	[*145*]
CD52	“	52	Alzetuzumab		*none*
CD79b	“	79b	Polatuzumab		*none*
GD2	disialogangloside 2		Dinutuximab, Naxitamab, Margetuximab	DB67	[*146*]
**Growth factors/receptor**					
a-beta	amyloid protein beta		Donanemab *	Ab-Apt, RNV95	[*147,148*]
CGRP	calcitonin gene-related peptide		Eptinezumab, Frenenezumab, Galcanezumab	Star-F12	[*149*]
HER2	human epidermal growth factor receptor 2		Trastuzumab, Pertuzumab, Margetuximab	HB5, HeA2_3	[*150,151,152,153*]
PDGFR-a	platelet-derived growth factor receptor alpha		Olaratumab	PDR3	[*154*]
VEGF	vascular endothelial growth factor		Bevacozumab, Ranibizumab, Brolucizumab	SL2b, many more	[*155,156*]
VEGFR2	vascular endothelial growth factor receptor 2		Ramucrumab	Apt01, 02, NX1838	[*157,158*]
Factor IXa/X	X coagulation factor IXa/X		*5*Epicizamab	9.3t, RB006	[*159*]
vWF	Von Willebrand factor		Caplacizumab	ARC1779	[*160*]

## Data Availability

The data presented in this study are available on request from the corresponding author. The data are not publicly available because of privacy restrictions.

## Supplementary Materials

The following supporting information can be downloaded at: https://www.mdpi.com/article/10.3390/ijms24043525/s1, including experimental results of nucleotidic TeTCIs targeting thrombin and SARS-CoV-2 RBD proteins, and methodology employed to locate and choose the references. References [51,175] are cited in the supplementary materials.

## Abbreviations

ADE:	adverse drug effect
AFS:	aryl-fluorosulfate (aryl-OSO_2_F)
bioTCI:	biomolecular targeted covalent inhibitor
CD:	cluster of differentiation
CHO:	Chinese hamster ovary
CS:	complimentary strand
CuAAC:	copper(I)-catalyzed azide-alkyne cycloaddition
Da:	Dalton
DS:	double strand
EdUTP:	5-ethynyl-dUTP
EGFR:	epidermal growth factor receptor
ELISA:	enzyme-linked immunosorbent assay
Fab:	fragment antigen binding (variable)
FasL:	Fas ligand (CD95L, CD178)
Fc:	fragment crystallizable (constant)
FcR:	Fc receptor
FDA:	Food and Drug Administration (USA
GPCR:	G-protein-coupled receptor
HER2:	human epidermal growth factor 2
mAb:	monoclonal antibody
NK:	natural killer
NGS:	next-generation sequencing
OctdU:	octadiynyl-dU
PCR:	polymerase chain reaction
PD1:	programmed cell death protein 1 (CD279)
PD-L1:	programmed death-ligand 1 (CD274)
PK:	pharmacokinetic
PPI:	protein-protein interaction
PS:	phosphorothioate
RBD:	receptor binding domain
SARS-CoV-2:	severe acute respiratory syndrome-associated coronavirus-2
SELEX:	Systematic Evolution of Ligands by EXponential enrichment
S_N_2:	bimolecular nucleophilic substitution
S_N_Ar:	nucleophilic aromatic substitution
SuFEx:	sulfur (VI) fluoride exchange
SDS:	sodium dodecyl sulfate
TBA:	thrombin binding aptamer
TCI:	targeted covalent inhibitor
TeTCI:	tethered targeted covalent inhibitor
TRAIL:	TNF-related apoptosis-inducing ligand
Uaa:	unnatural amino acid
