# Supplementary material for: bioTCIs: Middle-to-Macro Biomolecular Targeted Covalent Inhibitors Possessing Both Semi-Permanent Drug Action and Stringent Target Specificity as Potential Antibody Replacements
Source: Int J Mol Sci. 2023 Feb 9;24(4):3525. doi: 10.3390/ijms24043525 (PMC9968108; doi:10.3390/ijms24043525)
Supplement: Supplementary file 1 [file ijms-24-03525-s001.zip › ijms-2153550-supplementary.pdf]

Supplementary figures:

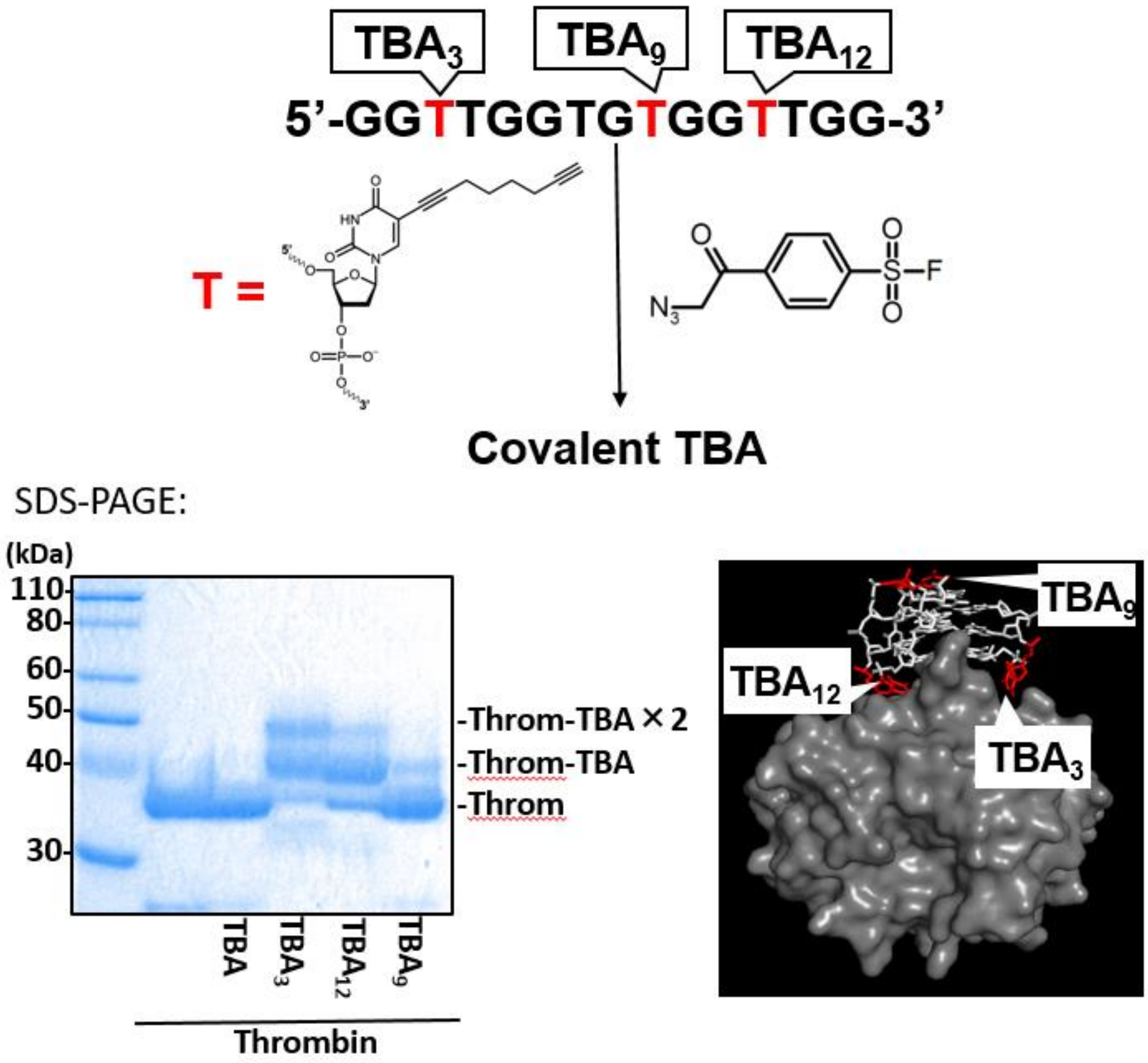

Figure S1 *Nucleotidic tethered-TCI (TeTCI) targeting thrombin.*
(A) An ocadinyl-dU replaced the T residue at the 3, 9, or 12th position of the 15-mer thrombin binding aptamer (TBA). A 4-(azidoacetyl)-benzene-1-sulfonylfluoride warhead was incorporated by the CuAAC reaction creating the TBA3, 9, or 12 aptamer TeTCIs. (B) A SDS-PAGE showing thrombin without or with the TeTCI. TBA is the unmodified 15-mer. Note the mobility-shifted thrombin bands when reacted with TBA3~TBA12>>TBA9 consistent with a covalent attachment of the TeTCI to the target protein. (C) A docking model showing the relative positions of the three TeTCIs bound to the thrombin target. This figure is modified from Tabuchi *et al.*, *Chem Commun* **2021**, *57*, 5378.

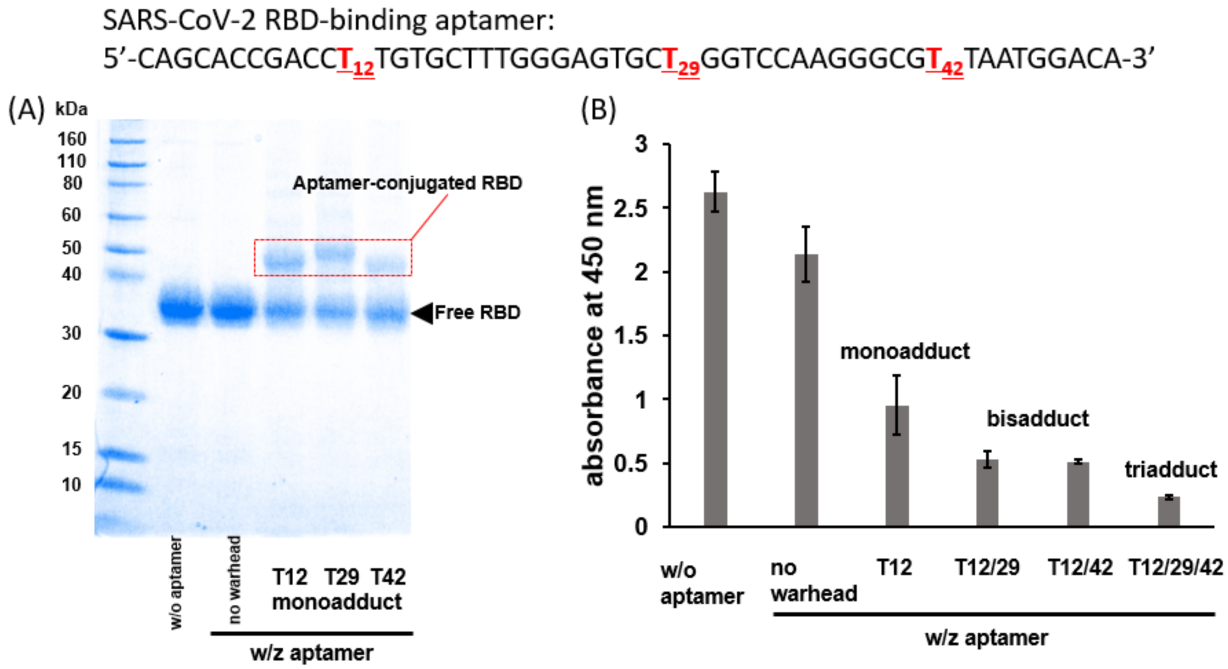

Figure S2 *Nucleotidic tethered-TCIs (TeTCIs) targeting SARS-CoV-2 S-protein receptor binding domain (RBD).* The 51mer SARS-CoV-2 RBD-binding aptamer (Song *et al.*, *Anal Chem* **2020**, *92*, 9895) was modified by replacing T12, T29, or T42 with octadiynyl-dU, and the 4-(azidoacetyl)-benzene-1-sulfonyl fluoride warhead was introduced by the CuAAC reaction creating the T12, T29, or T42 TeTCIs (top). Simultaneous multiple warhead introduction at these positions was performed in the same manner. (A) *TeTCI-protein conjugation.* SDS-PAGE of the RBD protein with the respective TeTCIs (i.e., warhead-monoadducts) showed a mobility-shift consistent with the covalent attachment of the TeTCI to the target. (B) *Target-protein inhibition.* ELISA of the RBD interaction with angiotensin converting enzyme 2 protein (RayBio Inc, COVID-19 Spike-ACE2 binding assay kit II) showed TeTCI inhibition of the protein-protein interaction (PPI). T12/29, T12/42, and T12/29/42 indicate the TeTCIs endowed with multiple warheads. TeTCIs possessing multiple warheads showed greater inhibition that the T12 monoadduct (unpublished data).

Methodology used to locate and choose the references cited:

Both PubMed and SciFinder databases were searched with keywords including antibody replacements, mAb therapy, covalent drugs, targeted inhibitors, peptide drugs, peptidomimetic drugs, aptamers, SELEX. The collected papers were discussed between the authors, and the final cited papers were selected. Clinical trials literatures on TCIs, even those leading to FDA approval, were beyond the scope of our review and not included, since potential mAb replacement was the theme.
